# Supplementary material for: Release of Plasmodium sporozoites requires proteins with histone-fold dimerization domains
Source: Nat Commun. 2016 Dec 16;7:13846. doi: 10.1038/ncomms13846 (PMC5172368; doi:10.1038/ncomms13846)
Supplement: Supplementary Information — Supplementary Figures 1-9, Supplementary Table 1, Supplementary Methods and Supplementary References. [file ncomms13846-s1.pdf]

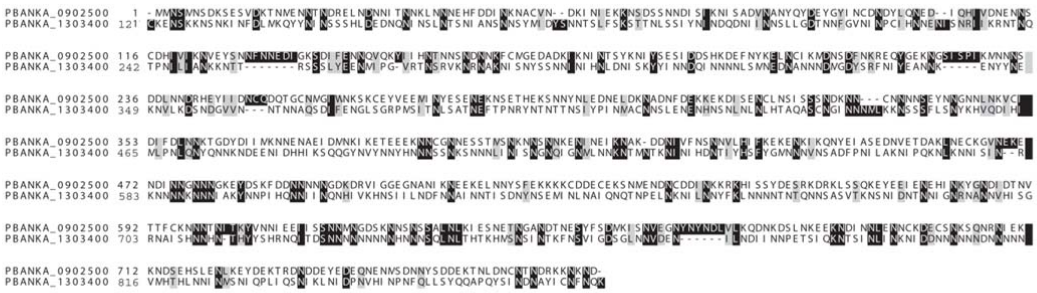

Supplementary Figure 1. Alignment of ORP1 and ORP2 (PBANKA_0902500 and PBANKA_1303400). The HFDs were excluded from the alignment, and included aa 1-720 of PBANKA_0902500 and aa 121-755 of PBANKA_1303400. Notable is the high content of asparagine in both proteins (24 and 31% respectively). The alignment was obtained using ClustalW2.

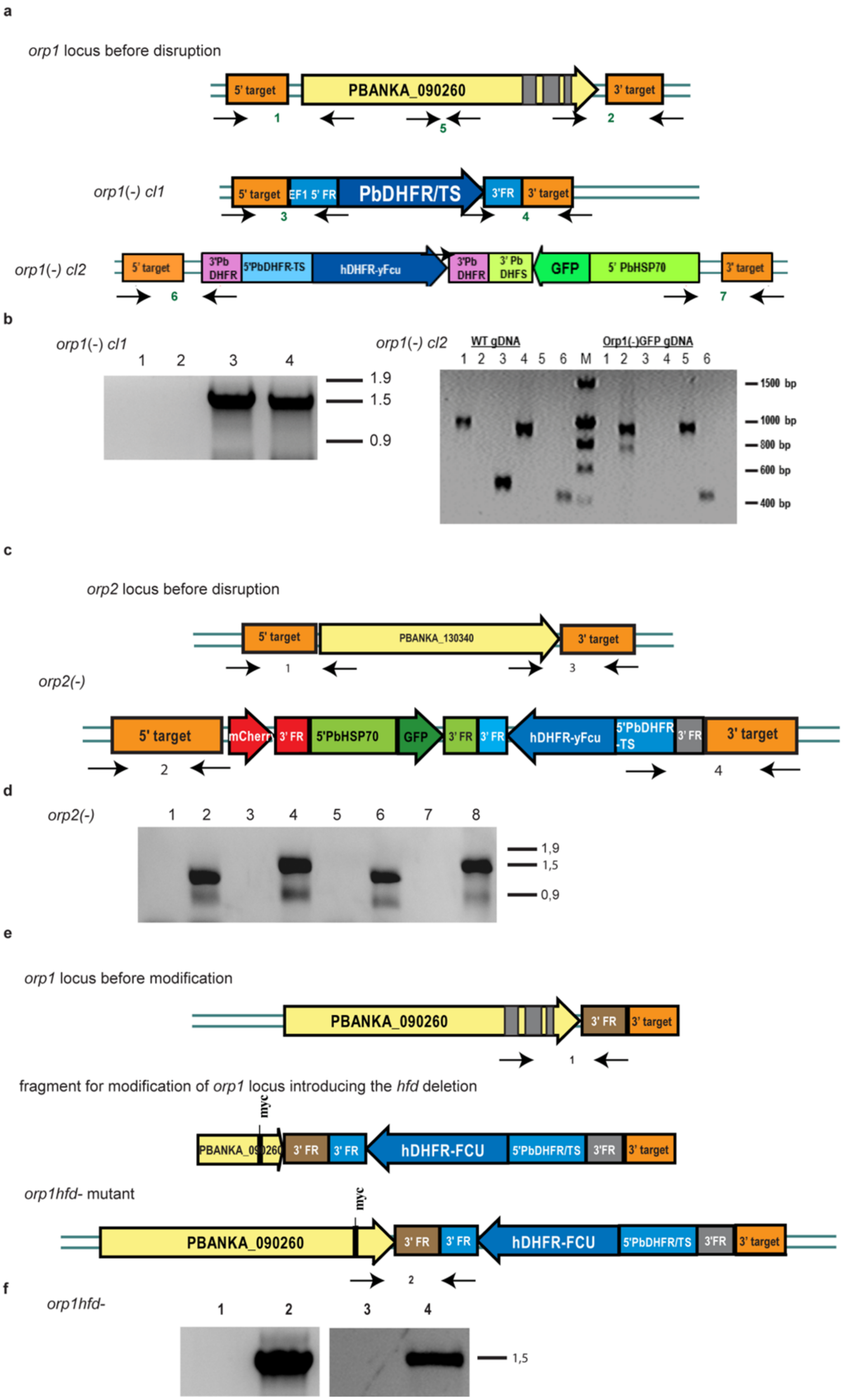
a
orp1 locus before disruption
5' target
PBANKA_090260
3' target
1
5
2
orp1(-) cl1
5' target
EF1 5' FR
PbDHFR/TS
3'FR
3' target
3
4
orp1(-) cl2
5' target
3'Pb DHFR
5'PbDHFR-TS
hDHFR-yFcu
3'Pb DHFR
3' Pb DHFS
GFP
5' PbHSP70
3' target
6
7
b
orp1(-) cl1
1 2 3 4
1.9
1.5
0.9
orp1(-) cl2
WT gDNA
Orp1(-)GFP gDNA
1 2 3 4 5 6 M 1 2 3 4 5 6
1500 bp
1000 bp
800 bp
600 bp
400 bp
c
orp2 locus before disruption
5' target
PBANKA_130340
3' target
1
3
orp2(-)
5' target
mCherry
3' FR
5'PbHSP70
GFP
3' FR
3' FR
hDHFR-yFcu
5'PbDHFR-TS
3' FR
3' target
2
4
d
orp2(-)
1 2 3 4 5 6 7 8
1,9
1,5
0,9
e
orp1 locus before modification
PBANKA_090260
3' FR
3' target
1
fragment for modification of orp1 locus introducing the hfd deletion
myc
PBANKA_090260
3' FR
3' FR
hDHFR-FCU
5'PbDHFR/TS
3'FR
3' target
orp1hfd- mutant
myc
PBANKA_090260
3' FR
3' FR
hDHFR-FCU
5'PbDHFR/TS
3'FR
3' target
2
f
orp1hfd-
1 2 3 4
1,5

Supplementary Figure 2. Construction and genotyping of mutants. a,b, Construction and genotyping of *orp1(-)*.**a**, Top: Schematic representation of the WT PBANKA_0902500 locus (exons are shown in yellow, introns in grey). 5' and 3' targeting fragments are indicated in orange. Middle: For the *orp1(-) cl1* mutant the gene was replaced via a double cross-over integrating the *PbDHFR/TS* cassette encoding anti-folate resistance (blue colour). Bottom: For the *orp1(-) cl2* mutant the same genomic regions were targeted as in *orp1(-) cl1*, but the insertion comprised the drug selectable cassette *hDHFR* fused to yeast *Fcu* (blue) and the gene encoding GFP under the control of the HSP70 promoter (green). The primers used for the PCR analysis are indicated as arrows, where the beginning of the arrow corresponds to the primer binding site. **b.** Genotyping of the *orp1(-)* mutants using PCR. The template was derived from *orp1(-)* gDNA. Left: For *orp1(-) cl1* WT contamination was tested with primer pairs 260-Dx-INT/260-Dx-EST (primer pair 2, expected size 1459 bp) and 260S-EST/260S-INT (primer pair 1, expected size 1572 bp) (lane 1 and 2 respectively). Primer pairs used for amplification of the right region of the mutant gene were DHFR-3525-A and 260-Dx-EST (primer pair 4, expected size 1624 bp) and 260S-EST and DHFR-2165-B (primer pair 3, expected size 1549 bp) (lane 3 and 4 respectively). Molecular weight markers are indicated to the right in kb. Right: Genotyping of WT and *orp1(-) cl2* . WT contamination was tested with primer pair 260S-EST/260S-INT2 (lane 1, expected size 1018 bp), TFseq4-for/Tfseq4rev, expected (primer pair 5, lane 3, expected size 510 bp) and 260Dx-INT2/260Dx-EST (lane 4, expected size 956 bp). Primer pairs 260S-EST/3' PbDHFR-bk (primer pair 6, lane 2, expected size 950 bp), were used to detect left integration and promHSP70-bk/260Dx-EST (primer pair 7, lane 5, expected sized 970 bp) right integration. Primer pair 0623200for/0623200rev amplifying a fragment from the control gene PBANKA_0623200 (expected size 402 bp) were used for quality control of the gDNAs. **c,d** Generation and genotyping of *orp2(-)*. **c,** Schematic representation of the WT locus (*orp2* ORF in yellow) and the modified locus of *orp2(-)*. 5' and 3' targeting fragment (orange) are indicated for integration via double crossover of a fragment containing the drug selectable cassette *hDHFR* fused to yeast *Fcu* (blue) and the gene encoding GFP under the control of the HSP70 promoter (green). Additional sequences of the plasmid vector pBATSIL6 are indicated in red (mCherry) and grey (3' flanking region, FR). The primers used for the PCR analysis are indicated as arrows, where the beginning of the arrow corresponds to the primer binding site. **d**, Genotyping of *orp2(-)* using PCR. Lanes 1, 3, 5 and 7: WT; lanes 2 and 4: *orp2(-) cl1*; lanes 6 and 8: *orp2(-) cl2*$^-$. Lanes 1 and 5: WT contamination was tested using primers 130340-sin-est with 130340-sin-int (expected size 1342 bp). Lanes 2 and 6: Integration of the 5' targeting fragment was analysed using primers 130340-sin-est with mCherry-back expected to give a 1200 bp fragment in the mutant. Lanes 3 and 7: WT contamination was tested using 3'UTR 130340D-ext2 with 130340Dint; (expected size 904 bp). Lane 4 and 8: Integration of the 3' targeting fragment was tested with primers 130340D-ext2 with 5'PbDHFR-back with a theoretical size of 1771 bp. **e,f,** Construction and genotyping of *orp1hfd*$^-$. **e,** Schematic illustration of the genomic WT locus of PBANKA_0902500 (top, ORF in yellow, introns grey), the construct used for gene replacement (middle) and the modified locus (bottom). The construct comprised of the last 890 bp of the first exon of the gene, a 25 bp region encoding c-myc in frame with the last 270 bp of the coding region followed by a stop codon and 595 bp of the 3'-FR (brown). A 597 bp fragment downstream of the 3'-FR of *orp1* was used as a 3' targeting sequence (orange). The drug selectable cassette encoding hDHFR fused to yeast Fcu is indicated in blue and a 3'FR fragment of the vector pBATSIL6 in grey. Integration of the construct by double cross-over resulted in 189 bp deletion of the *orp1* ORF encoding part of the α2 to αC domains of the HFD (amino acids 797 to 860). The primers used for the PCR analysis are indicated as arrows, where the beginning of the arrow corresponds to the primer binding site. **f**, The mutant genotype of each clone was confirmed using used primers Diagn-sin-for and ORP1(myc-fine)-rev to detect WT contamination (lane 1 and 3), (expected size 1569 bp); and primers Diagn-sin-for and hDHFR-B to detect the mutant clones: *orp1hfd*$^-$ *cl1* (lane 2) and *orp1hfd*$^-$ *cl2* (lane 4), (expected size 1413 bp).

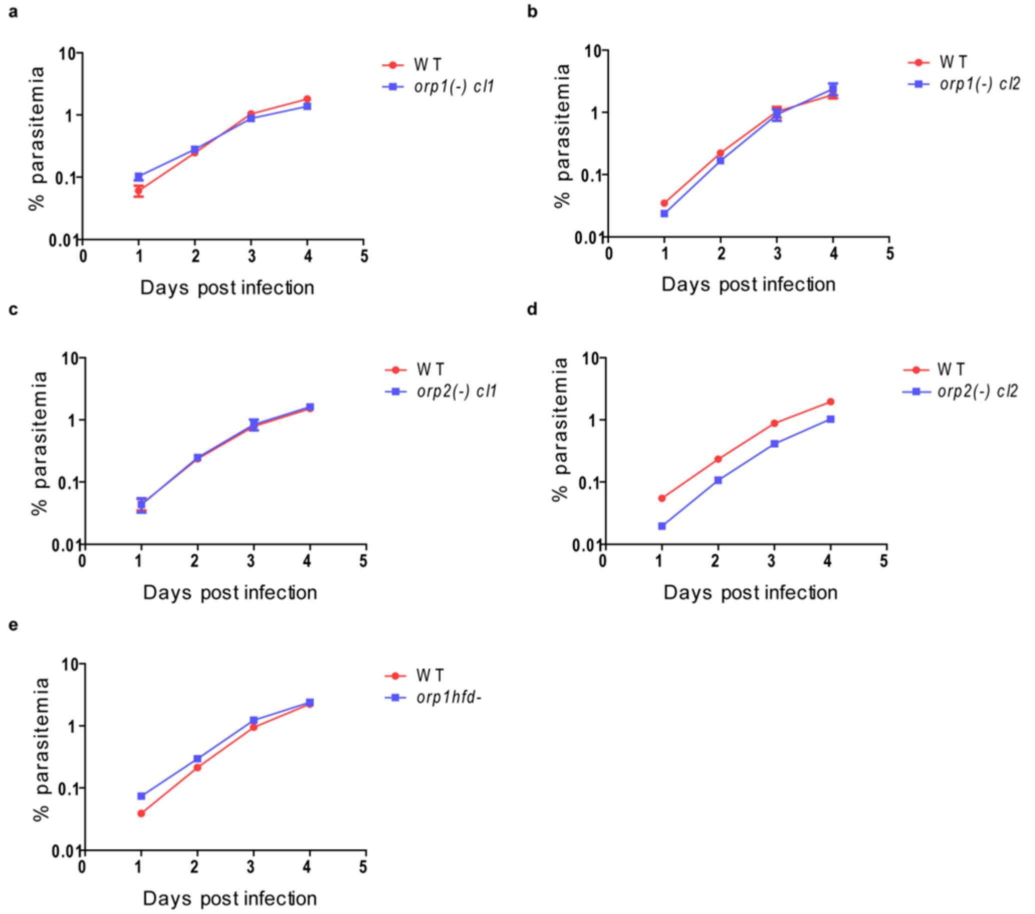

Supplementary Figure 3. Blood stage growth curves of mutants. a, Comparison of growth of WT and *orp1(-) cl1*.A mixture of red blood cells infected with WT (strain HP-GFP) and *orp1(-) cl1* were injected in the tail vein of two CD mice. The sample was stained with Hoechst 33342 and $5x10^5$ cells counted in the cytofluorimeter counting separately GFP positive and negative cells. **b,** Comparison of growth of WT and *orp1(-) cl2*. A mixture of red blood cells infected with WT (strain HP) and *orp1(-) cl2*, which is GFP positive, were injected in the tail vein of two CD mice. The samples were processed as in a. **c, d,** Comparison of growth of WT and *orp2(-) cl1* and *cl2*. A mixture of red blood cells infected with WT (strain HP) and *orp2(-)* clones, which are GFP positive, were injected in the tail vein of two CD mice. The samples were processed as in a. **e,** Comparison of growth of WT and *orp1hfd*$^-$ *cl1*. A mixture of red blood cells infected with WT (strain HP-GFP) and *orp1hfd*$^-$ were injected in the tail vein of two CD mice. The samples were processed as in a. All graphs show mean values of duplicate experiments ±s.e.m.

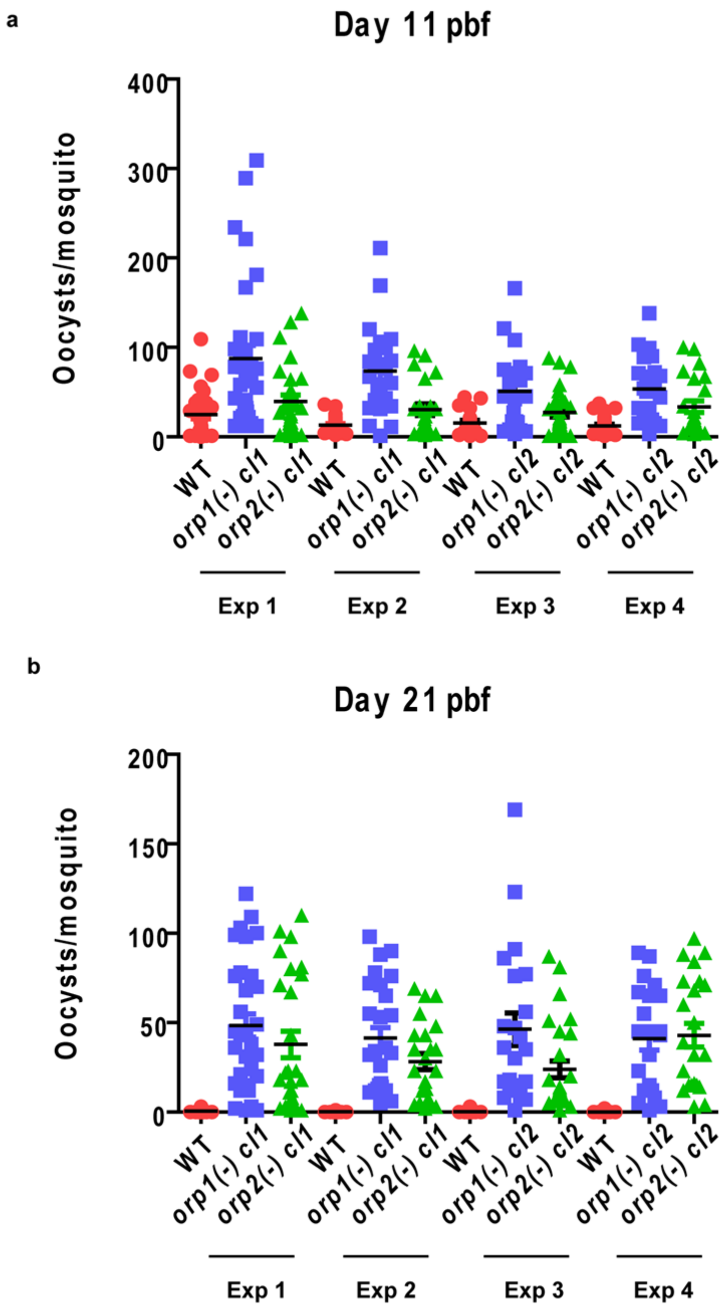

Supplementary Figure 4. Oocyst loads of disruption mutants at day 11 and 21. a, Oocyst loads of WT (red circles), *orp1(-) cl1* and *cl2* (blue squares) and *orp2(-) cl1* and *cl2* (green triangles) counted at day 11 pbf. **b**, Oocyst loads of WT (red circles), *orp1(-) cl1* and *cl2* (blue squares) and *orp2(-) cl1* and *cl2* (green triangles) counted at day 21 pbf. The complete dataset and the P-values (Mann-Whitney test) are presented in Supplementary Table 1. Bars denote mean ±s.e.m.

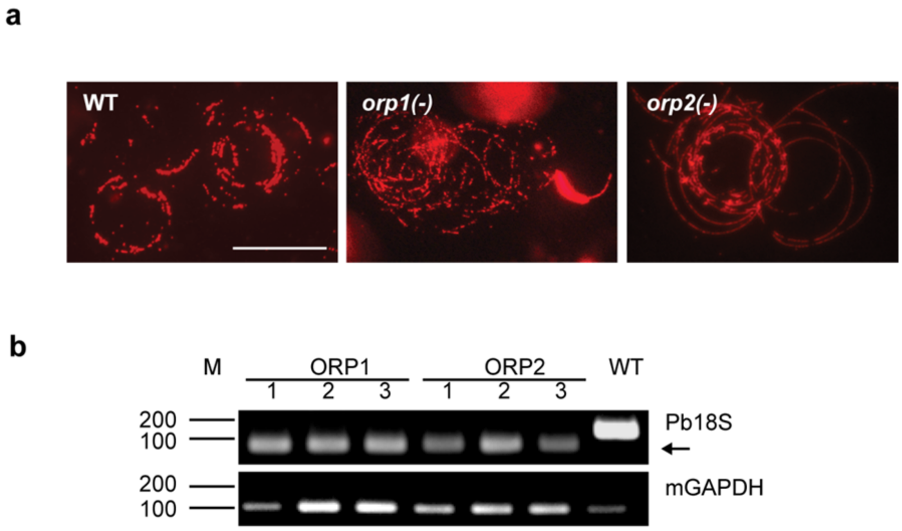

Supplementary Figure 5. Mutant sporozoites are motile but do not establish an infection in the mouse liver. a, Sporozoite motility. WT (left), *orp1(-) cl1* (middle) and *orp2(-) cl1* (right) sporozoite motility visualized as CSP labelled trails. Oocysts were mechanically ruptured to release sporozoites which were allowed to glide on glass slides, followed by CSP antibody labelling. WT sporozoites were from day 15 oocysts, and mutant sporozoites from day 17. The circular trails are indicative of motility. In the central panel a brightly labelled sporozoite is visible. Scale bar, 25 µm. **b,** RT-PCR analysis of livers collected from mice injected with OPR1 (n=3), ORP2 (n=3) and wild-type (WT n= 3 pool) parasites at 44 hours post-infection. Transcripts were detected by PCR, using primers specific for Pb18S gene and mouse glyceraldehyde-3-phosphate dehydrogenase (GAPDH) as a control. The lower band in the upper panel is primer dimer (arrow). See also Supplementary Methods.

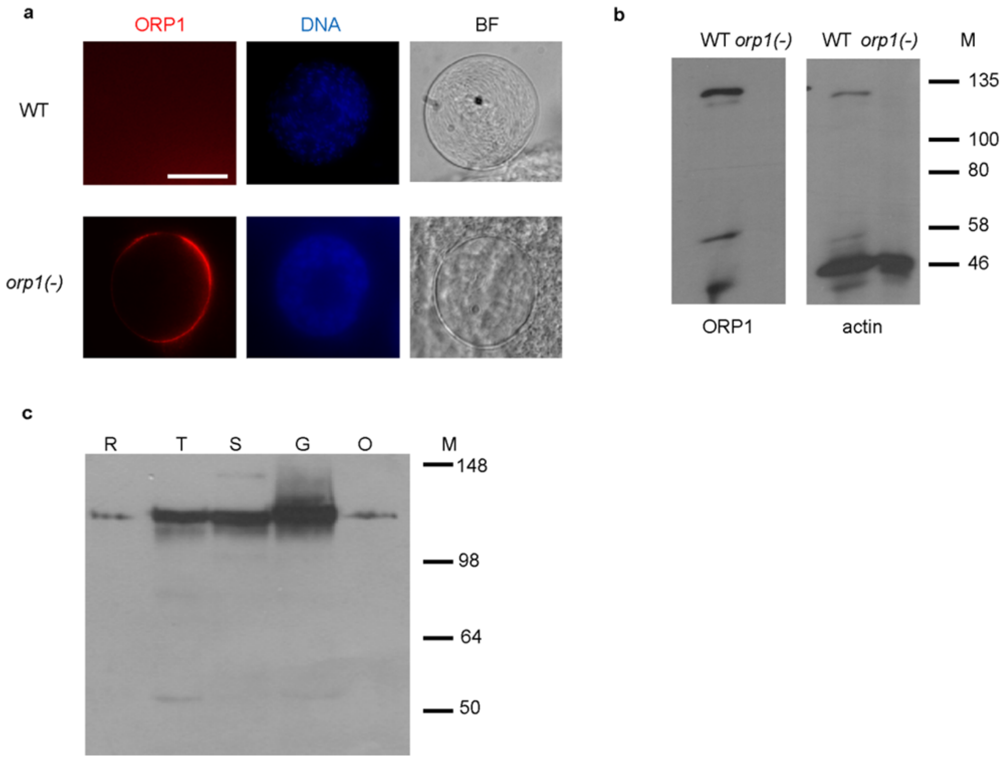

Supplementary Figure 6. Validation of the antibody recognizing ORP1. a, WT (top) and *orp1(-) cl 1* (bottom) oocysts 12 days pbf were immunolabelled with the antibody against ORP1 (red). DNA was stained with DAPI (blue), BF=bright field. Scale bar, 15 µm. **b,** Western blot of WT and *orp1(-)* ookinetes. Left: The antibody against ORP1 recognizes a high molecular band of the expected molecular weight (calculated molecular weight 110 kDa) only in the WT sample. A smaller fragment at weaker intensity was also visible (~ 50 kDa), suggesting a processed form. Right: As a loading control the blot was re-probed with a monoclonal antibody against actin (molecular weight 42 kDa), specific for the parasite[1]. As both antibodies are recognized by the same secondary antibody the ORP1 bands are also visible on this blot. M, molecular weight markers. **c,** Western blot probed with the ORP1 antibody. Samples loaded were synchronized asexual stages (R, ring stages; T, trophozoites; S, schizonts), gametocytes (G) and ookinetes (O). M, molecular weight markers. See also Supplementary Methods.

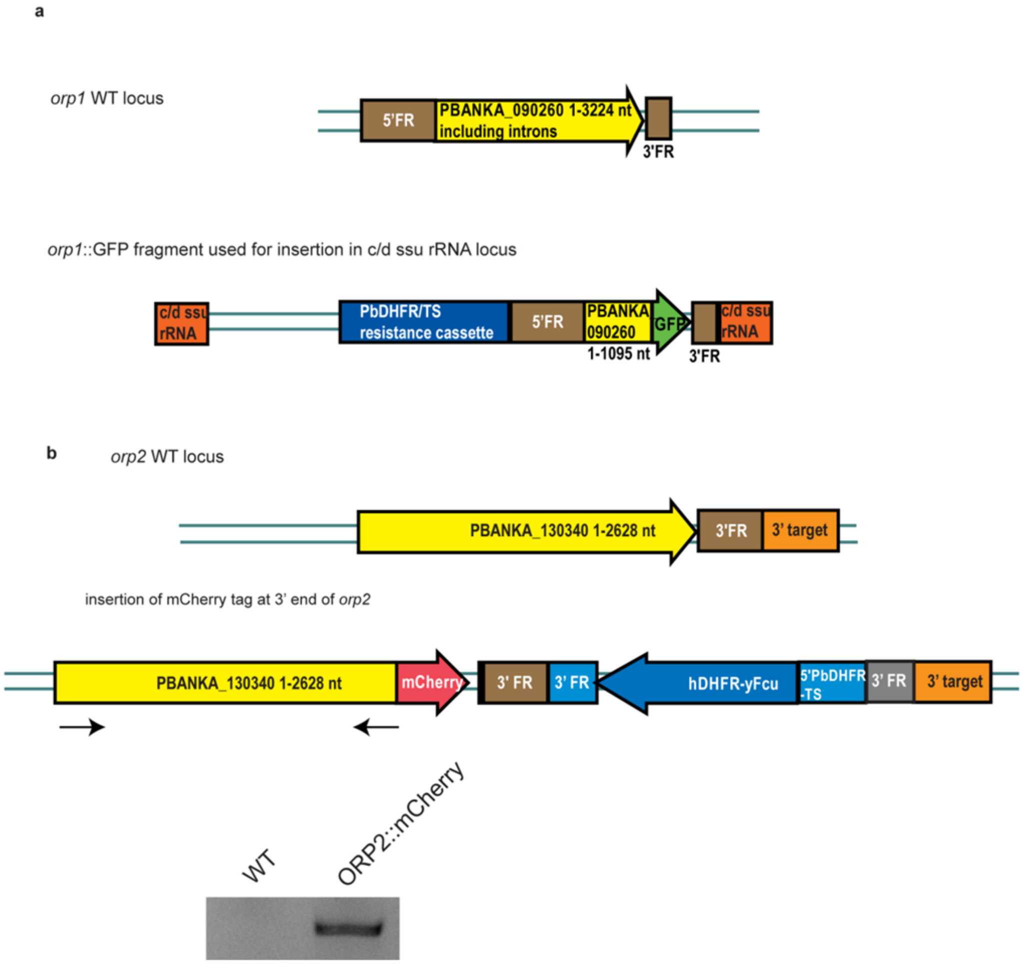

Supplementary Figure 7. Construction and genotyping of *orp1::GFP* and *orp2::mCherry*. a, Schematic representation of the ORP1 construct comprising the first 1095 bp of the ORF (yellow) fused in frame to the gene encoding GFP (green). 5' and 3' FRs (brown) were derived from the *orp1* locus. The plasmid contained targeting sequences (orange) derived from the C ssu-rRNA locus and the construct was introduced in the C/D ssu-rRNA locus via a single cross over. **b**, Schematic representation of the *orp2* WT locus (ORF in yellow, top) and the modified locus (middle). A double crossover strategy was used to insert an mCherry coding gene (red) in frame to the 3'end of the *orp2* coding region (yellow) followed by the 3'FR of the gene (brown). The drug selectable cassette encoding hDHFR fused to yeast Fcu is indicated in blue and the 3' targeting fragment in orange. The primers used for the PCR analysis are indicated as arrows, where the beginning of the arrow corresponds to the primer binding site. The correct insertion was verified by PCR using primers 130340S1 and mcherry-back (bottom). Note that scale is different in a and b.

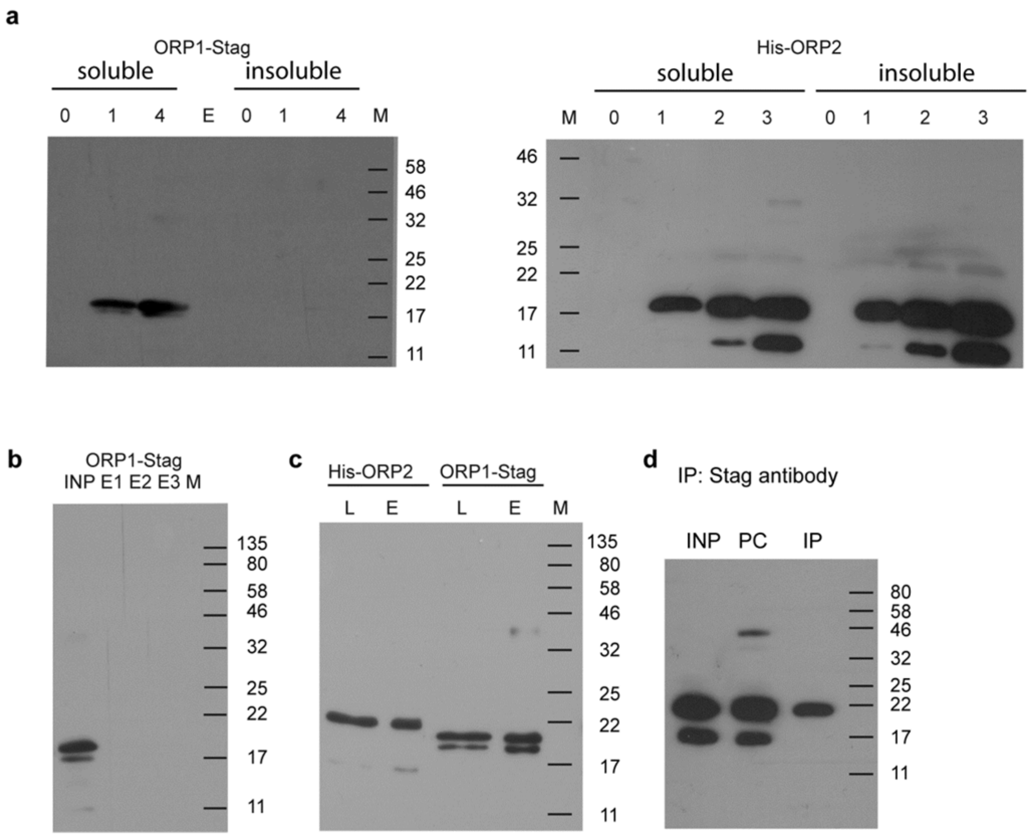

Supplementary Figure 8. ORP1-Stag is not retained on Ni-NTA in the absence of His-ORP2. **a,** ORP1-Stag (left) and His-ORP2 (right) were expressed as soluble proteins in E. coli. Shown are samples obtained at different time points after induction of expression with IPTG. The samples were separated in soluble and insoluble fraction by centrifugation after sonication. **b,** ORP1-Stag was expressed in E. coli transformed with pET-Duet-1 containing only the ORP1 HFD. A crude lysate (lane 1) was mixed with Ni-NTA beads using the stringent conditions described in Fig. 4. The samples loaded in lanes E1-E3 are three consecutives eluates from the beads. **c,** The complete Western blot of pull-down experiment shown in Fig. 4 b. **d,** The complete Western blot of the co-immunoprecipitation shown in Fig. 4 c. M, molecular weight markers in kDa.

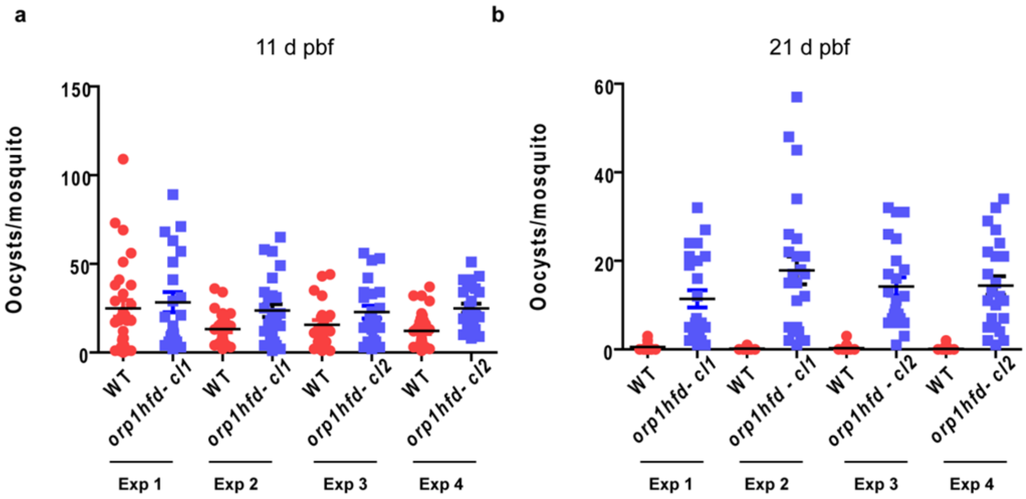

Supplementary Figure 9. Oocyst loads of *orp1hfd*$^-$ mutant clones at day 11 and 21. a, Oocyst loads of WT (red circles) and *orp1hfd*$^-$ *cl1* and *cl2* (blue squares) counted at day 11 pbf. **b**, Oocyst loads of WT (red circles) and *orp1hfd*$^-$ *cl1 cl1* and *cl2* (blue squares) counted at day 21 pbf. The complete dataset and the P-values (Mann-Whitney test) are presented in Supplementary Table 1. Bars denote mean ±s.e.m.

Supplementary Table 1. Summary of mosquito infections of WT and *orp1(-)*, *orp2(-)* and *orphfd*$^-$ mutants.

Exp.	Strain	Parasitemia (%)[a]	Gametocy-temia (%)[a]	Exflagella-tion[b]	Ookinete conversion (%)[c]	Oocysts/ midgut (range) d 11[d]	P (n)[e]	Oocysts/ midgut (range) d 21[f]	P (n)[g]	Midgut spz d 11 x10[3h]	Midgut spz d 21 x10[3i]	Salivary gland spz d 21 x10[3] (n)[j]
1	WT	6.8	0.80	6.6	52	25 (0-109)	(30)	<1 (0-3)	(31)	3.1	0	3.9 (31)
	orp1(-) cl 1	5	0.35	2.6	32	87 (12-309)	P<0.0001 (31)	48 (1-122)	P<0.0001 (32)	5.2	4.9	0 (33)
	orp2 (-) cl 1	6.2	0.67	6	37	37 (1-138)	P=0.14 (28)	38 (1-110)	P<0.0001 (26)	4.1	7.8	0 (24)
	orphfd$^-$ *cl 1*	7.5	0.80	5	44	28 (2-89)	P<0.0001 (22)	11 (1-32)	P<0.0001 (30)	3.6	3.4	0 (22)
2	WT	8.2	0.76	4	45	13 (2-36)	P=0.5408 (29)	<1 (0-1)	30	3.8	0	4.2 (30)
	orp1(-) cl 1	6.5	0.70	4	49	74 (1-211)	P<0.0001 (25)	41 (5-98)	P<0.0001 (26)	3.5	4.3	0 (26)
	orp2 (-) cl 1	6.8	0.67	6	37	30 (2-96)	P=0.0057 (22)	28 (2-69)	P<0.0001 (23)	4.3	4.0	0 (22)
	orphfd$^-$ *cl 1*	5.2	0.5	4	42	24 (1-65)	P=0.0543 (26)	18 (1-57)	P<0.0001 (24)	3.6	3.5	0 (23)
3	WT	5	0.4	3	54	16 (1-44)	(22)	<1 (0-3)	22	3.9	0	4 (22)
	orp1(-) cl 2	5	0.4	3	38	51 (3-166)	P=0.0024 (22)	47 (1-169)	P<0.0001 (22)	4.5	4.1	0 (22)
	orp2(-) cl 2	6	0.4	3	42	27 (1-88)	P=0.0606 (28)	24 (1-87)	P<0.0001 (27)	3.9	4	0 (25)
	orphfd$^-$ *cl 2*	6.7	0.7	4	46	23 (2-56)	P=0.1417 (22)	14 (1-32)	P<0.0001 (22)	3.6	2.4	0 (22)
4	WT	8.3	0.8	5	47	12 (1-37)	(31)	<1 (0-2)	29	3.7	0	4.2 (29)
	orp1(-) cl 2	8	0.8	5	47	46 (3-138)	P<0.0001 (21)	41 (1-89)	P<0.0001 (21)	4.8	4.7	0 (21)
	orp2(-) cl 2	7.8	0.7	4	49	34 (3-100)	P=0.0054 (23)	43 (3-97)	P<0.0001 (23)	4.2	4.1	0 (24)
	orphfd$^-$ *cl 2*	7.6	0.7	4	47	25 (8-51)	P=0.0001 (23)	15 (1-34)	P<0.0001 (23)	3.0	3.0	0 (23)

Four experiments were carried out each with WT and the three mutant strains. [a] Percentage of parasitemia and gametocytemia was determined on Giemsa stained blood smears. [b] Exflagellation was counted under the microscope after dilution of infected blood in RPMI and incubated at 19°C for 15 minutes. [c] Female gamete to ookinete conversion was determined after labelling overnight *in vitro* ookinete cultures with an antibody against the surface protein Pbs21 and counting elongated ookinetes and round female gametes and zygotes under the microscope. [d] Oocysts were counted on midguts dissected from infected mosquitoes 11 d pbf. Range of values is indicated in parenthesis. [e] P values of pairwise comparisons of oocysts counts of WT compared to each mutant using the Mann-Whitney test. n=Number of midguts counted. [f] Oocysts were counted on midguts dissected from infected mosquitoes 11 d pbf. Range of values is indicated in parenthesis. [g] P values of pairwise comparisons of oocysts counts of WT compared to each mutant using the Mann-Whitney test. n=Number of midguts counted. [h] Midgut sporozoites/mosquito from homogenized samples. Number of midguts is the same as in column [e]. [i] Midgut sporozoites/mosquito from homogenized samples. Number of midguts is the same as in column [g]. [j] Salivary gland sporozoites/mosquito, n=number of dissected mosquitoes.

Supplementary Methods

Sporozoite gliding motility.

Glass slides were coated with RPMI with 3% bovine serum albumin (BSA). Midgut sporozoites were added to the slides and incubated for 60 min at 37°C, then fixed with 4% paraformaldehyde, and labelled with the CSP antibody[2].

RT-PCR analysis of liver infection

C57BL/6 mice were injected with midgut sporozoites collected from mosquitoes 18 days post-infection and the mice were killed 44 h later. Livers were removed, homogenized, and subjected to RNA isolation via Trizol (Ambion) with cDNA synthesized by SuperScript III First-strand Synthesis System (Invitrogen) as per manufacturer's instructions. PCR was conducted by amplification of parasite ribosomal 18S rRNA gene using the primes forward 5'-TTTATTGGGAGATTGGTTTTGACGTTTATGTG-3' and reverse 5'-AAGCATTAAATAAAGCGAATACATCCTTAC-3' (117bp). As a control mouse GAPDH gene was amplified by forward primers 5' – CAACTCCCACTCTTCCAC-3' and reverse 5'-CTGTAGCCGTATTCATTGTC-3' (99bp). Thermo cycling conditions were 1 cycle of 95 °C for 5 min followed by amplification of the target cDNA for 35 cycles of 95 °C for 30sec and 55 °C for 30sec.

Western blot analysis.

Enriched samples of different stages of the life cycle were analyzed by Western blot after separating the proteins on an 8% SDS-PAGE gel. The blot was probed with the antibody directed against ORP1, followed by anti-rabbit antibodies conjugated with horse radish peroxide (Jackson Immunoresearch). The membrane was developed using the ECL system (SuperSignalWest Pico, Thermo Scientific) according to manufacturer's instructions.

Supplementary References

1 Westphal, M. *et al.* Microfilament dynamics during cell movement and chemotaxis monitored using a GFP-actin fusion protein. *Curr Biol* **7**, 176-183 (1997).

2 Potocnjak, P., Yoshida, N., Nussenzweig, R. S. & Nussenzweig, V. Monovalent fragments (Fab) of monoclonal antibodies to a sporozoite surface antigen (Pb44) protect mice against malarial infection. *J Exp Med* **151**, 1504-1513 (1980).
